# Romani Women and Health: The Need for a Cultural-Safety Based Approach

**DOI:** 10.3390/healthcare10020271

**Published:** 2022-01-30

**Authors:** Fernando Jesús Plaza del Pino, Oscar Arrogante, Juana Inés Gallego-Gómez, Agustín Javier Simonelli-Muñoz, Gracia Castro-Luna, Diana Jiménez-Rodríguez

**Affiliations:** 1 Department of Nursing, Physiotherapy and Medicine, University of Almeria, 04120 Almería, Spain; sma147@ual.es (A.J.S.-M.); graciacl@ual.es (G.C.-L.); d.jimenez@ual.es (D.J.-R.); 2 Red Cross University College of Nursing, Spanish Red Cross, Autonomous University of Madrid, 28003 Madrid, Spain; oscar.arrogante@cruzroja.es; 3 Faculty of Nursing, Campus de los Jerónimos s/n., Catholic University of Murcia, 30107 Murcia, Spain; jigallego@ucam.edu

**Keywords:** women, Romani, health, cultural safety, cultural competence

## Abstract

The Romani are the main European ethnic minority. The Romani people’s situation of social vulnerability and their difficulties accessing the health system make their health indicators worse than those of the non-Romani population. The present study will delve into health beliefs, and experiences with health services and professionals, through the perspectives of Romani women. In this qualitative study, 16 women of different ages were interviewed in a city located in the South of Spain. Four themes emerged from the analysis of the data: the construction of the identity of Romani women, difficulties in life, health and disease beliefs and barriers to accessing the health system. We conclude that every project for the improvement of the health of the Romani community must take into account the active participation of Romani women and must consider the principles of Cultural Safety, by delving into the intercultural training of health professionals and addressing the social determinants of health which affect the Romani collective.

## 1. Introduction

The Romani (gypsy) population is the main European minority in Europe. Spain has the largest Romani population in Western Europe: approximately 800,000 individuals. The autonomous community with the largest population of Romani is Andalusia, with an estimated population of 290,000 individuals, followed by Catalonia, with 90,000, Madrid with 70,000, and the Valencian Community with an estimated population of 58,000 Roma people [1]. The Romani collective is very diverse [2]; age, sex, economic status, place of residence, family, type of formal education, type of employment, ideology, religious beliefs, etc., can have an influence on the degree of identification of each person with what could be denominated “Romani identity” and the daily reality in which they live.

In a comparative study on the situation of the Romani population in Spain, related to employment and poverty [3], we find some interesting data that could help us address the reality of this collective. The Spanish Romani population is very young as compared to the non-Romani population; 45% are younger than 16 years old, and the rate of birth is 64 per 1000, while for the non-Romani population, it is 14 per 1000. As for the level of education, only 17% of the Romani population has finished Mandatory Secondary Education, as compared to 77% of the general population; in the case of Romani women, this decreases to 15.5%. The rate of illiteracy is 14% for the female population, and 6% for the male population. In this study, it was also shown that, with respect to employment, the Romani men had an employment rate of 44%, while the women had an employment rate of 17%, much lower than the mean for women in the general population, which is 44%.

Romani women neither study nor work, with 58% dedicated to domestic work. In fact, the main reason for the inactivity of Romani women is associated with family responsibilities, which also demotivates them from seeking employment [3]. The role of Romani women within their community for leading changes that improve the living conditions of their families should be highlighted [4]. Romani women are still suffering from inequality, as evidenced in numerous studies and reports. Sexism affects all women similarly, but Romani women also suffer from social and cultural discrimination due to the existing racism against them. Due to this sexism and racism, they have more problems accessing and participating in education processes, a reality that has negative repercussions as it perpetuates their situation of exclusion [5]. It is believed that Romani women suffer from a triple exclusion: gender, ethnic origin, and for having, in most cases, a low level of education [4]. The type of position that Roma women tend to occupy in the family also has an influence on the health inequalities, as they could be overloaded and more focused on the care of their family members than their own health [6].

Considering poverty thresholds, 66% of the Roma population suffers from severe poverty, and 86% of families are at risk of poverty [3]. The Romani population is one of the collectives that is currently most affected by exclusion, which, among other factors, is the result of historical processes of segregation, racism, and stereotyping of the Romani world [5].

A large percentage of Romani men and women conceive health as the absence of sickness, and sickness as an invalidating situation linked to death. This vision of health and sickness means that they are only concerned about health when symptoms begin to appear, so prevention measures are difficult to implement in this population [7,8].

The social determinants of health, “the circumstances in which people are born, work, live, and age, included in the broadest set of strengths and systems that have an influence on the everyday conditions of life” [9], mark the state of health of the Romani population, and are translated into health inequalities [10]. The Romani population is considered as a vulnerable group and has limited access to public health: exclusion and social marginalization processes limit the access or use by the Romani population of social health services directed towards the improvement of their living conditions [11]. The poor quality of health of the Roma population compared to the non-Roma population is generalized in all EU countries, mainly due to differences in the social determinants of health. The inequalities experienced by the Roma population in accessing quality education, the labor market, housing, health care and other public spaces can make a difference in people′s health [12]. A fact that evidences the significant differences between the state of health of the Romani population and the non-Romani one is that the life expectancy of the Romani population is seven years less than that of the general population [12].

The health of people is the result of a complex interaction between the determinants of health, opportunities to access health services, and the quality of experiences in health care [13]. Diverse studies [2,14,15] highlight the differences between Romani and non-Romani with respect to the barriers perceived in the access to health services, pointing to cultural differences, the prejudices and stereotypes of the health professionals, and the vulnerabilities of the Romani themselves as the most important factors to their access to health services. Both the unequal access to health services, as well as the social determinants of health, are avoidable and unjust, and contribute to health inequalities [6,16]. In the scientific literature, there is broad consensus with respect to placing the explanatory focus of health inequality of the Romani community on social determinants. Two large groups of factors are usually mentioned: those linked with social exclusion, and those associated with cultural elements. Both groups of factors are complementary, that is, no matter how much we address “the cultural”, it will be impossible to undo the inequalities without dealing with factors of social exclusion, and vice-versa [6].

Studies on the health of the Romani population from the point of view of the Romani themselves are very scarce. The scientific community itself has pointed to the exclusions created by studies which ignore the voices of the individuals studied, and whose final results increase the social exclusion of the studied population [17]. Macías and Redondo [5] highlight the important contribution of scientific research on overcoming the social exclusion of groups and collectives such as the Romani population, or, on the contrary, the effect it could have on the perpetuation of their situation of exclusion over time. It is necessary to increase the number of research studies to more adequately orient the social health policies that are focused on this collective [18].

In this sense, theoretical approaches based on the principles of Cultural Safety, which were proposed by the Maori nurse Irihapeti Ramsden [19], are adequate to improve the quality of healthcare services in intercultural settings. According to these principles, culturally safe practices are based on the recognition that we are all bearers of culture and, at the same time, we must be aware of and challenge social and health inequalities.

The objective of the present study is to delve into the perceptions of Romani women on their health beliefs and experiences with health services and their health professionals, as well as the role of Romani women in the health of their community.

## 2. Materials and Methods

A qualitative study was designed which followed an interpretative phenomenological approach as the best strategy for the understanding of human experiences [20]; studying an event, but from the perspective of those who experienced it, as people try to create meaning from their experiences [21]. In our case, we focused on the living conditions of Romani women, their traditions and cultural values with respect to health, and their experiences with health services and health professionals.

### 2.1. Participants and Context

The study informants were Romani women living in the south of Spain, and more specifically, in Almeria. An intentional sampling method was utilized, through the selection of participants of different ages, to ensure a broad exposure of the specific setting. All the women who participated had had considerable previous contact with the Public Health System. In addition, all participants lived in peripheral areas of the city with a medium-low socio-economic level, where there exist slums and where there is very deficient maintenance and cleanliness of the area. Their participation was voluntary.

### 2.2. Data Collection

The data collection was performed through semi-structured interviews, following a guide with a set of open-ended questions to facilitate the in-depth discussion of the subjects of interest.

The interviews were conducted during the month of October 2021, at the homes of the informants in order to establish an adequate environment to facilitate expressions of feelings and emotions in an atmosphere of sincerity. The mean duration of the interviews was forty minutes. The main researcher recruited the participants through non-governmental organizations working in the different neighborhoods, which provided the contact information of Romani women (RW) who met the inclusion criteria. The interviews were recorded with the participant’s permission. When 16 individuals were interviewed, the topics became repetitive. Therefore, the researchers considered that data collection had to stop, as data saturation had been reached [22], and, consequently, the data collection was considered complete.

### 2.3. Data Management and Analysis

The recorded interviews were transcribed and edited by the interviewer to guarantee the exactness of the transcription. The data were stored, managed, classified, and organized with the help of the analysis software ATLAS-ti 8.0. Firstly, an iterative reading of the transcripts was performed. The themes initially identified were aligned to the 4 open-ended questions formulated in each semi-structured interview. These questions were related to the participants’ perceptions about their Romany identity, social and economic conditions, health and sickness concepts, and experiences with health services. It should be noted that the theoretical framework to build these questions was based on the previous scientific literature and the objectives established in our study. Subsequently, the categories and their corresponding subcategories were identified according to the themes that emerged [23].

### 2.4. Validity and Reliability/Rigor

The reliability of the data was verified by comparing the codification by the three researchers who codified the transcripts (F.J.P.d.P., D.J.-R., and O.A.) independently. Subsequently, the consistencies and discrepancies among the three researchers were determined. The disagreements were resolved by consensus.

All the researchers were aware of their personal opinions and biases. The research team maintained a constant reflection throughout the process of analysis. The quotes that best represented the categories were extracted in order to present them in the results section.

The COREQ criteria were used as a guideline for reporting this qualitative study [24].

### 2.5. Ethical Considerations

The research protocol was approved by the Research Ethics Committee from the Department of Nursing, Physical Therapy, and Medicine from the University of Almeria (protocol number EFM 155/2021). To guarantee anonymity and confidentiality, a code was assigned to each participant. In all cases, the written informed consent form was provided to each of the RW, and an explanation of the form was provided.

## 3. Results

In the end, 16 interviews were given to RW. The mean age of the informants was 38.4 years old. Most of them were married, and the mean number of children was 4.8. As for their work, only five informants worked outside of the home, having to combine work with their main family caregiver role. Among these women, none had higher education, and half did not have any education. The characteristics of the informants are shown in Table 1.

After the initial identification of 32 codes from the data, other important codes emerged, which were grouped into categories and subcategories based on the four main themes that emerged from the questions asked. A more detailed analysis allowed us to identify categories and subcategories, which are shown in Table 2.

Next, for the presentation of results, we will expose the categories and subcategories of the study in order to simplify the results.

### 3.1. Construction of the Romani Woman Identity

All the RW who were interviewed were inclined to speak about what it meant to *be Romani*, their traditions and customs, and the role they played in their community.

- The pride of being Romani.

When the informants were asked about what it meant for them to *be Romani*, they mentioned their attachment to their traditions, their culture, the importance of family, and the respect and care of their elders.

*You don’t understand it because you are a “payo” (not Romani), for us being Romani is the most important thing, we have our traditions, and we like to carry them out.* RW13

*Among Romani, the elders are respected, we have them at home, the women in the family organize ourselves…the men, they better not become involved, they don’t know nothing (laughs).* RW6

- The role of the Romani woman.

As a mother, the RW is responsible for the care of her family, and this is expressed in such terms.

*Us Romani women take care of everything, the children, the house, and even the husband, when he is sick, we go to the doctor…we take care of everything, and when the market is open, we go to the market.* RW9

*The men are into other things, we are the ones who deal with the kids; it’s our tradition.* RW11

The Romani custom that the younger female child must remain single to be able to take care of the parents was obtained from the testimony of the informants.

*My mother has always been sick, I’m the youngest, and I remained single to take of her and my father…it’s like that…when they are no longer here, I’ll look for a husband (laughs).* RW12

The women feel responsible for transmitting the community values to their children, but are also aware that changes must be introduced that are oriented towards gender equality, as expressed by this woman:

*I make sure that my children are good Romani, and that what is ours is not lost (…) but all 4 children the same, I don’t want my girl to do as I did; all 4 children will finish school and high school, and when she is older, she will decide who she wants to marry.* RW16

### 3.2. Difficulties in Life

During the interviews, our informants mentioned the difficulties they found every day due to the lack of resources, unemployment, and job insecurity.

*My dad dedicated his life to metal scraps, he died very young, and we had to wake up to be able to feed ourselves.* RW5

*You don’t know what it is to wake up and go to the street and do whatever to be able to feed the children.* RW4

*My Manuel buys a box of fish at the port, and sells it in the neighborhood. If he’s successful, we’ll have enough to buy something, but if the police take it away, we are left empty-handed.* RW2

*With the (government) help, we have enough to eat, but we don’t have enough to pay for electricity and water.* RW1

### 3.3. Health and Sickness Beliefs

When delving into the health beliefs of RW, they demonstrated with their comments the relationship established between feeling healthy with being functional in their settings, to be able to keep on performing their responsibilities. They equated health with the absence of signs and/or symptoms of sickness.

- Being useful.

Our informants defined a direct relationship between their ability to play their role and their state of health.

*If you can’t continue with your responsibilities, it’s because something is wrong*. RW5

*Health is the most important thing, when you are healthy, you can deal with everything that is thrown at you.* RW3

- What you see.

For them, health, illness, severity or improvement is only reflected by symptoms. They showed some ignorance about the importance of prevention for health.

*My Antonio always had stomach pain, I would make him chamomile tea, and the pain went away, but then nothing worked and he had to be admitted, when he left (the hospital), he could live life as before, and the doctor would tell me that what he had was bad. How can it be bad if he was better with the medicines from the hospital?* RW9

*The nurse is always telling me that I have to lose weight, but I’m fine. What’s the point of being so skinny? (laughs)* RW10

### 3.4. Barriers in the Access to the Health System

The experiences of the RW with the health system and health professionals provided evidence about the difficulties they experienced in the access and use of health devices.

- Romani culture.

The responses highlight some situations of conflict from past hospital admissions, created by the lack of understanding of the health professionals about the importance they give to the company of family and the sense of *extended family* of the Romani culture.

*We are all about family, and when my Jose was admitted, everyone wanted to be with him, from the oldest to the youngest, it’s our custom (…), he was really proud about having his people with him (…), the truth is that they didn’t say anything to us, when going into the hospital, or being in the room, sometimes we had to leave the room when the nurses came to do something to him.* RW7

*One day the security guard came to make us leave, the truth is that we were a lot of people, but they have to come.* RW2

- Problems with the health professionals.

The prejudices against the Romani collective that are present in society were perceived by our informants in some of the health professionals. The RW explained to us that they felt a “differential” treatment from some health professionals, and that there was an existing lack of knowledge about the Romani culture.

*I remember a nurse who always seemed like she was angry, she didn’t speak to us much.* RW14

*It’s clear that the “payos” do not want us, always speaking badly about us, as if we didn’t notice how they look at us.* RW4

*Some are defensive with us, they probably think we will do something to them, or that we will make trouble.* RW3

*They do not understand how we Romani are, and how we do things.* RW15

## 4. Discussion

With the present study, we have approached the Romani culture, their beliefs in the area of health, and their experiences with the health system through interviews conducted with a group of RW.

The most common characteristics reflected in the participants of our study are: married women, with more than four children, low level of education, living in deteriorated areas of the cities, and with low levels of income, as shown in a previous study [3]. These hard life conditions determine their health.

The RW play a key role within their community as educators, caregivers of children and elders, transmitters of guidelines and values of the Romani culture, and promoters of change within their community, which makes them indispensable for the implementation and development of health programs for this collective [25].

Our results show a series of common cultural elements that form part of the identity of the Romani people, their own cultural identity that is not taken into consideration by the health services [26]. Values such as having an extended family or the view of sickness as the absence of health [7] are not recognized or correctly addressed, which creates conflicts and tense situations at health services [6,15]. The scarce amount of knowledge possessed by health professionals and the health system about the Romani people, their culture, and their beliefs about health, results in the inadequate health intervention for this collective with respect to their needs, and a lack of effectiveness of the services provided [27].

Our informants described diverse situations which showed the presence of prejudice and stereotyping by the health professionals against the Romani ethnic group, as other studies in the area of health have shown [15,28]. Studies conducted since the 1970s have persistently evidenced that this sector of society has suffered the greatest attitudes of rejection [29], and the most discrimination in our society [30], which perpetuates the negative treatment of this collective [31]. The problems in their relationships with health professionals are yet another set of barriers to accessing to the health system which need to be addressed.

We believe that the incorporation of Romani intercultural mediators and Romani health professionals will help establish cultural bridges between Romani and non-Romani people. This will improve the cultural knowledge of the health professionals and relationships in the area of health, weakening existing prejudices and stereotypes, as shown in different studies with the Romani collective [15] and other minor ethno-cultural groups [32,33].

As it is very well known, the health of the Romani collective is marked by their situation of social vulnerability and their living conditions. In order to improve the health of Romani women and men, it is indispensable to address the social determinants of health—scarce resources, low level of education, unhealthy living environments, etc.—along with the difficulties accessing health care due to cultural differences and the prejudices of professionals [2,34].

It is therefore necessary to address the health of the Romani collective from a perspective that recognizes the Romani cultural identity and questions the inequalities of power that exist in the relationships at individual, as well as family, community and social levels, and that is willing to defy them. The best model for this challenge is Cultural Safety [19], which has been demonstrated to have great efficacy in decreasing inequalities in the health of vulnerable groups and in improving the socio-sanitary conditions of these populations for which it has been applied, at the same time as it opposes the view that the only thing needed for the care of culturally-differentiated groups is the cultural competence of the health professionals [35]. We must visualize the reality of a Romani minority, promote actions from the health system and public health to address their health needs, and address the social and political determinants of their exclusion (all with the active participation of Romani men and women) to be able to move forward towards social equity and better health of the Romani population [36].

The health and/or socio-sanitary intervention programs with the Romani community must take into account the principles of Cultural Safety: (1) Protocols that determine the respect of the cultural customs of the individual and the group, developed with the active participation of the individuals and groups involved, (2) personal knowledge and training of professionals, (3) a process that ensures the participation of the community, (4) a positive guaranteeing that the process obtains the most adequate and positive result for the target of the service, in agreement with his or her values, preferences, and way of life, and, lastly, (5) alliances between professionals and the individuals and/or groups involved [37].

### Limitations

Although we achieved homogeneity in the sample, our findings may not be able to be applied to the entire Romani collective, although they can bring us closer to the RW’s own view about their role in the community and their relationship with the health system. We believe that the participation of more Romani informants from different environments could help us obtain a broader view of our object of study. In addition, we have yet to delve into the situation of the RW from a perspective of gender, an objective for future studies.

## 5. Conclusions

The present study presented an emic perspective of the RW about their own role in the community and their relationship with the health system. Our results highlight the important role of the RW as caregivers and transmitters of values, which makes their active participation indispensable in the design and development of health programs to be implemented in the Romani collective.

Intercultural training of health professionals that is centered on the overcoming of prejudices and stereotypes, increasing knowledge and understanding of the Romani culture and values, and in the acquisition and/or improvement of communication skills is needed.

The interventions in the health and socio-sanitary areas of the Romani collective must follow the principles of Cultural Safety.

## 6. Implications for the Practice

As a differentiated cultural group, the care of Romani individuals needs a comprehensive assessment that takes into account their values, health beliefs, and customs. The care of the Romani patient needs cultural adaptation, and greater efforts towards the improvement of communication and the relationship with the patient and the family.

In the area of hospitals, solutions need to be found that are agreed upon between the institution, health professionals, and families, to avoid conflicts, with respect to visits and stays at the center.

## Figures and Tables

**Table 1:** Characteristics of the informants.

Code	Age	Children	Work	Education	Marital Status
RW1	24	3	Home	Primary	Married
RW2	36	4	Home	Secondary	Married
RW3	23	3	Home	Secondary	Married
RW4	28	2	Home	Primary	Married
RW5	43	5	Home and cleaner	No education	Separated
RW6	51	5	Home	No education	Widow
RW7	52	4	Home	No education	Married
RW8	29	2	Home and street vendor	Primary	Married
RW9	47	4	Home and street vendor	No education	Widow
RW10	32	3	Home	Secondary	Married
RW11	31	3	Home	Primary	Married
RW12	19	0	Caring for parents	No education	Single
RW13	62	7	Home and street vendor	No education	Widow
RW14	58	6	Home	No education	Married
RW15	35	3	Home and cleaner	Primary	Married
RW16	45	4	Home	No education	Married

**Table 2:** Table of Thematic categories.

Themes	Categories	Sub-Categories
Romani identity	Construction of the Romani woman identity	Pride of being Romani
		The role of the Romani woman
Social and economic conditions	Difficulties in life	Living conditions
Health and sickness concepts	Health and Sickness beliefs	Being useful
		What you see
Experiences with health services	Barriers in the access to the health system	Romani culture
		Problems with health professionals

## Data Availability

The data presented in this study are available on request from the corresponding author.
